# Framing the education for gifted Lebanese and gifted refugees in Lebanon

**DOI:** 10.3389/fpsyg.2022.1077278

**Published:** 2022-12-13

**Authors:** Maya Antoun

**Affiliations:** Department of Education, Faculty of Arts and Sciences, University of Balamand, Beirut, Lebanon

**Keywords:** conceptions, professional learning, gifted refugees, identification, model, gifted education

## Abstract

The paper provides a comprehensive conceptual model or framework for the identification and programming of gifted education in Lebanon. The model is evidence-based on the critical review of the literature on gifted education over the past two decades. The model discussed topics related to teacher conceptions, identification, preparation, and practices for the gifted in Lebanon. The development of a culturally appropriate conceptual model of gifted education is locally focused on Lebanese teachers’ needs to identify and serve gifted Lebanese and gifted refugee learners. This framework provides a means for educational leaders in Lebanon to consider policy reforms that will benefit not only gifted learners but also gifted refugee learners in different educational settings.

## Introduction

Although people of all races and cultures have talents and abilities, not everyone has access to the same resources and supports in life (Lee et al., 2022). Gifted children are sometimes overlooked, concealed, or ignored. Therefore, there is a critical need to increase access to gifted education by identifying and developing the hidden potential of all gifted students, including those from underprivileged and refugee groups who might be less likely to receive such identification and support in Lebanon. Even unintentionally, traditional academic research can perpetuate stigma and power disparities. As educators and researchers, our role is geared toward research that can be applied to confront inequities and advocate for the rights of gifted students who have diverse needs, values, opportunities, and experiences (Ford, 2014).

Al-Hroub (2022) reported that most Arab nations view the education of the gifted as essential to the growth and prosperity of their country. Yet Lebanon still does not seem to be adequately supporting students who are gifted and talented (Lebanese Association for Educational Studies (LAES), 2006; Sarouphim, 2010; Al-Hroub, 2022; Antoun et al., 2022). However, adopting approaches that have been developed for other cultural contexts may not be an appropriate response. Without research that is specific to the Lebanese context, the western view will be all that is available to influence the process of identification and education of the gifted. As argued by Khaleefa (1999), the “practice of importing methods of studying creativity, intelligence and giftedness without rigorous adaptation is handicapping for Arab research in these concepts” (p. 25). Although this point was raised almost two decades ago, little has changed in terms of understanding giftedness within the Lebanese context. Educational leaders need to utilize the potential within their student body. Enhancing the potential of every student through innovative curriculum and improved teacher education underpins each nation’s responsibilities within its education system. It would appear that investing in a system of education that challenges students to think critically and creatively, which are underpinning tenets of gifted education, could be particularly helpful in a country that is experiencing a range of problems requiring new and innovative solutions.

## The Lebanese context

Education in Lebanon is considered a major priority for the Lebanese people and a central pillar of the Lebanese economy and society (Abdul-Hamid and Yassine, 2020). The educational sector has been one of Lebanon’s most prominent distinguishing features (Vlaardingerbroek et al., 2017). Despite that, the reality of student learning outcomes shows that the country is experiencing a learning crisis, with low overall levels of learning and high levels of inequality. Since 2007, there has been a downward trend in student performance, with Lebanon performing poorly when compared to other nations (Abdul-Hamid and Yassine, 2020; Antoun et al., 2023). In addition to low levels of learning, Lebanon has significant levels of inequality. The learning outcomes gap between the top and bottom economic, social, and cultural status quintiles is the greatest in the Middle East and North Africa region (Abdul-Hamid and Yassine, 2020).

According to Frayha (2009), the Lebanese education system is currently facing a major problem of quality disparities between schools. Lebanese schools provide different learning experiences and education standards. In Lebanon, private and public schools offer various qualities of education. Students from disadvantaged socioeconomic backgrounds attend public schools known for their reputation for poor educational quality (Bahous and Nabhani, 2008; Ayyash-Abdo et al., 2010; Bahou, 2015; Antoun et al., 2020) while private schools, known for their high-quality education, have been sought by the middle and high-income groups. In Lebanese schools, Arabic, English, and French are the three main languages taught. Each school’s language of instruction is either English or French and is used to teach all subjects, except for the Arabic language and social studies (Vlaardingerbroek et al., 2017).

In Lebanon, the gifted population is marginalized as special needs programs primarily target pupils who struggle in the classroom. There are no funds allocated to support and develop gifted learners in public schools (Al-Hroub, 2022). The potential of high-achieving students is nurtured in some private schools that cater to children from the upper socioeconomic class by providing enrichment programs (Sarouphim, 2009, 2015; Al-Hroub, 2022). These programs, however, lack the theoretical foundation of those provided for talented students in western institutions, as there are no regulations governing the provision of services to gifted and talented learners (Sarouphim, 2009). The rhetoric of the Lebanese Ministry of Education appears to focus on the same endpoint for all students. This is achieved through provisions being made for those with disabilities, to help bring their achievement up to an acceptable level, without considering the needs of highly able students, who are already well above average (Sarouphim, 2010, 2015; El Khoury and Al-Hroub, 2018).

## Refugee and underrepresented gifted students in Lebanon

Colonial practices have affected Lebanon’s educational system and since Lebanon’s independence in 1943, they have disenfranchised a sizable portion of the vulnerable Lebanese children enrolled in public schools. Children from neglected and underprivileged communities in Lebanon had poor education. Due to the low tuition costs, the majority of low socioeconomic class families in the country choose to enroll their children in public schools and the recent overlapping crises have had disastrous effects on their children. Numerous groups of gifted children are disregarded and marginalized, such as gifted refugees, gifted students from low socio-economic backgrounds, and twice-exceptional children.

Lebanon, a small heterogeneous country, has historically welcomed hundreds of thousands of refugees and migrant workers. Thousands of Syrian and Palestinian refugees currently reside in Lebanon. Prior to the current economic crisis, it was already challenging for Lebanon to meet the requirements of vulnerable people and refugees. Children from refugee backgrounds encounter a wide range of difficulties that prevent them from receiving an education that meets their unique requirements (Khansa and Bahous, 2021). Students who are Palestinian refugees in Lebanon encounter obstacles and problems in exercising their unalienable right to an education, and they require support to have more and simpler access to educational possibilities. Lebanese public schools only accept a small number of these children, and many of them cannot pay the tuition at private schools (Al-Hroub, 2014b; Shuayb, 2014). Most Palestinian students attend UNRWA (United Nations Relief and Works Agency) schools, where they follow the Lebanese curriculum. Poor socioeconomic conditions, a lack of financial aid and scholarships, restrictions on attending Lebanese public schools, and inadequate instruction and facilities in UNRWA schools are just a few of the difficulties these refugee students are facing.

Syrian students are another group of students who make up a sizable portion of the student body in Lebanese public schools. The Lebanese government opened its public schools and offered afternoon shifts to Syrian refugees (Mendenhall et al., 2017). Lebanese curriculum was to be used exclusively for Syrian refugee students’ education in terms of curriculum and instruction (Abdul-Hamid and Yassine, 2020). Although the Lebanese government welcomed Syrian refugees into its educational institutions, it fell short of giving them a quality education since in Syria all subjects are taught in Arabic (Immerstein and Al-Shaikhly, 2016). For Syrian students, studying subjects in a foreign language is considered a barrier to their academic success (Shuayb et al., 2014; Mendenhall et al., 2017). Research data revealed the inequality faced by marginalized students in Lebanon because of their poor schooling experiences (Shuayb, 2014; Bahou, 2015; Al-Hroub, 2020; Al Khalili, 2021). Key findings by Al-Hroub (2014b) and Shuayb et al. (2014) showed that the education provided for Syrian refugees was falling short due to the inflexibility of the curriculum and the absence of additional academic support to facilitate their transition to the new learning environment, resulting in high levels of dropout rate.

High academic performance becomes more difficult to achieve when students experience unequal access to educational opportunities (Reardon and Portilla, 2016), teacher bias (Nicholson-Crotty et al., 2016), and limited resources (Olszewski-Kubilius and Corwith, 2017). Students from refugee backgrounds need specialized programs that are designed to meet their unique academic, linguistic, cultural, and social demands (DeCapua et al., 2009). Research showed that there is a lower likelihood of identifying gifted children among disadvantaged children, minority populations, second-language learners, and gifted children with disabilities (Card and Giuliano, 2016; Wright and Ford, 2017; Gentry et al., 2019; Ricciardi et al., 2020). Inequities in access to and opportunity for high-quality education start at the time of entry into school and lead to lifelong inequities in educational achievement for many children and adolescents who live in low-income households and are economically marginalized (Barrett et al., 2019). According to Proctor et al. (2019), gifted children from underprivileged backgrounds have enormous potential, but they are systematically discriminated against because they attend institutions with inadequate resources and low-quality curricula. Missed chances lend credence to allegations of elitism in gifted programs. In other words, these marginalized students fail to meet the traditional criteria for identification. Research showed that underrepresentation might be a result of misconceptions, teacher bias, as well as the use of screening techniques (e.g., nonverbal tests to identify non-native English speakers) that are inappropriate (Hamilton et al., 2020). Researchers in gifted education advise employing a variety of techniques to identify individuals for gifted programs, such as alternative assessment measures, and multiple criteria adoption (Ecker-Lyster and Niileksela, 2017; Dixson et al., 2020; Lee et al., 2022).

## Lebanese teachers’ conceptions and identification procedures

Communities may have different values, and attributes prized in some countries may not be valued to the same extent in others (Freeman, 2005; Sternberg, 2007). Lebanese people tend to place a high premium on affluence, appearances, and social prestige with a focus on achievement as an end product. This view is supported by Al-Hroub (2022) and Ayyash-Abdo et al. (2009), who concur that the emphasis of the Lebanese curriculum is on academic subjects with achievement favored. The culture and environment in which Lebanese teachers work and live have an impact on how they view giftedness and the educational opportunities available to gifted students. According to Bevan-Brown (2005) and Antoun et al. (2022), differences in culture have an impact on the identification and learning processes, as well as behavior standards and classroom interactions. Misconceptions about giftedness can impact identification and educational provision. For example, within Lebanon, the idea of giftedness seems to fit better with achievement, specifically highlighting the value of effort and underplaying natural ability, when in reality, natural ability and different catalysts are important components of gifted development. As a result, it is unlikely that elements like gifted underachievement and twice exceptionality would be taken into account in the context of the overall picture of giftedness (Antoun et al., 2022). Sarouphim (2009) contends that the concept of giftedness in Lebanon is equal to high academic performance. Similarly, Antoun et al., 2020 revealed that many Lebanese teachers perceive gifted learners as those who excel in the academic subject matter. However, many Lebanese teachers also consider social intelligence, leadership, and creativity as special areas of giftedness (Al-Hroub and El Khoury, 2018). Thus, high intelligence could be misinterpreted and used interchangeably with the concept of giftedness (Al-Hroub, 2010a).

As pointed out by Kibbi (2003), Lebanese culture has little tolerance for deviation from tradition and it has been argued that Lebanese people have been encouraged to obey, compromise, and accept social obligations. In Lebanon, an emphasis is placed on mastering and perfecting skills through rigid training (Kibbi, 2003; Ayyash-Abdo et al., 2009; Sarouphim, 2009). As highlighted in the previous literature, academic achievement is highly valued in the Lebanese culture (Kibbi, 2003; Ayyash-Abdo et al., 2009; Bacha and Bahous, 2011) which affects identification and provision. Findings from Antoun’s (2022) study describe how teachers’ attitudes and educational provision in the classrooms appear to have been influenced by Lebanon’s broader socioeconomic and cultural background. A few Lebanese studies contributed to the literature in terms of understanding teachers’ perceptions and provision in relation to the needs of their gifted students. Antoun et al. (2020) highlighted how Lebanese primary school teachers focused on the intellectual component of giftedness rather than on a broader conceptualization. The primary teacher participants placed more emphasis on the intellectual aspect of giftedness than on a broader conception. The study’s teacher participants linked academic aptitude with giftedness and stressed the value of nurturing students’ gifts in relation to traits that might be categorized as behavioral or personality traits. Lebanese teachers placed a higher value on math and science and equated giftedness with high academic achievement. In line with prior research (Renzulli, 2003; Dweck, 2006; Pfeiffer, 2013), the study emphasized deliberate practice and effort as ways for learners to achieve their maximum potential. However, only a small proportion of the teacher participants acknowledged the impact of assessment practices on performance by high-achieving and gifted learners, thereby revealing a lack of knowledge of the idea of gifted underachievement, which assumes that individuals with excellent reasoning skills might not be driven to reach their potential to highly achieve (Antoun et al., 2020). According to the study, Lebanese primary schools’ identification procedures mainly rely on teacher observation, which is *ad hoc* and based on a conception of giftedness that promotes performance above the growth of high potential (Antoun et al., 2020). While Gagné (2003, 2004) clearly distinguished between intellectually gifted and academically talented students, this distinction is something that is not understood among Lebanese teachers. Sarouphim (2009) points out that in Lebanon, academic success in disciplines like math and science is the primary criterion used to assess aptitude.

Al-Hroub (2022), Sarouphim (2010, 2015), and Diab (2006) stated that the country lacks effective methods for identifying giftedness, with Diab (2006) noting that all intelligence tests utilized in Lebanon are imported mainly from the United States and France. Thus, these measures used in Lebanon provide only a rough estimate of the student’s ability as they mainly measure cognitive ability, leaving a need for reliable and valid procedures for identifying gifted Lebanese and non-Lebanese students, including refugees.

According to Sarouphim (2009), Lebanon desperately needs research on identifying measures. She consequently investigated how well the performance-based assessment DISCOVER identified, talented Lebanese students. Her research revealed that teachers in Lebanon believed they knew more about their students’ skills than any test could measure. Since all identified students had excellent averages, she was able to uncover a solid match between teacher nominations and DISCOVER ratings, confirming teachers’ belief that academic excellence and giftedness are equivalent concepts. Her study led to the proposal that teachers and parents receive training on how to recognize and support their children’s giftedness.

## Instructional practices

Kibbi (2003) and Frayha (2003) confirm that teaching and learning within Lebanese classrooms follow mostly traditional instructional methods and are carried out formally where active and collaborative learning is absent and is not given enough importance. The growth of creativity can be negatively impacted by a lack of emphasis on developing reasoning skills and encouragement of conformity. School-based teaching strategies prevent learners from voicing their ideas, criticizing, or engaging in argumentative behavior in favor of memorization and rote learning (Al-Haj, 1995; Bahous et al., 2011), in opposition to recommended practices in gifted education. According to Maker and Shiever (2010), the development of a learning community and the facilitation of changes in content, method, and product adjustments require a shift away from the instructor as the authority. Teachers, rather than serving as information providers, take on the roles of coaches and facilitators that influence talent development (Gagné, 2008; Antoun et al., 2022). Most models for educating the gifted advocate the use of the constructivist approach, whereby instruction focuses on problem-solving, creativity, and exploration (Maker et al., 2006; Al-Hroub and Krayem, 2018). As a result, such an approach to education could represent a significant prototype shift in Lebanon, given the fact that teachers often play the position of knowledge producer (Frayha, 2003).

According to research, teachers are not effectively catering to highly able/gifted students (Lebanese Association for Educational Studies (LAES), 2006; Sarouphim, 2010; Krayem and Al-Hroub, 2019; Antoun et al., 2022). The belief that intellectually gifted students would succeed regardless of the type of educational setting in which they are placed is the root of such resistance (Antoun et al., 2020). These views are in opposition to research, which shows that all students need to be intellectually challenged and that developing a student’s gifts and abilities requires effort and the right provision and accommodation.

A study by Al-Hroub and El Khoury (2018) also revealed that services and governmental programs for the gifted were said to be almost nonexistent. Sarouphim (2010, 2015) believes that a fundamental lack of understanding of the idea of giftedness is a major contributing factor to Lebanon’s lack of progress in gifted education. It was also evident from another Lebanese study data provided by primary teacher participants that there was a lack of recognition by the Lebanese teachers that gifted students had wide-ranging abilities which could be developed into talents in primary school (Antoun et al., 2020). Data also showed that a substantial effort was put into the identification of the building blocks (i.e., hard work, and persistence) within Lebanese schools. However, this has not necessarily translated into providing the effective type of educational provision that is promoted in the literature (Shavinina, 2009; Rindermann and Ceci, 2018; Subotnik et al., 2019). Results of a mixed-method case study that examined the opinions of more than 280 Lebanese teachers about methods for identifying and teaching highly able/talented primary school pupils revealed that instructors had concerns about providing specialized services for gifted students (Antoun et al., 2022).

## Professional knowledge

Within Lebanon, there are no unified standards for teacher preparation and education (Bahous et al., 2011). Although Lebanon is in great need to rely on the contributions of its elite generation and most able minds, information on how to educate gifted and talented students in the nation is scarce for teachers. Teachers offer one-size-fits-all curricula rather than adjusting their instruction to the needs of particular students. Teacher preparation programs primarily concentrate on mainstream education (Sarouphim, 2010). As a result, the country lacks many teachers who are certified in gifted education.

It has been proposed that the perceptions and self-efficacy of instructors regarding their ability to provide appropriate educational opportunities for gifted students are impacted by their lack of special education preparation (Matheis et al., 2019). Plunkett and Kronborg stated that teachers’ professional development is essential for overcoming misconceptions that result from a lack of teacher education or from low confidence in their ability to meet the academic needs of exceptional children (Plunkett and Kronborg, 2011, 2019).

Researchers in gifted education have found that gifted provision should match the gifted student’s unique needs, interests, and abilities (Gallagher, 1985; Knight and Becker, 2000; Al-Hroub, 2010b; Antoun et al., 2022). Al-Hroub (2022) stated that there is a lack of qualified teachers to assist gifted learners at all levels. A study by Antoun et al. (2023) investigated the reasons for a few Lebanese students have been able to perform at the highest level in mathematics and science in TIMSS, by studying how the national Policy documents and the mathematics and science centralized curricula and textbooks address the needs of highly able students. Teachers’ perceptions and role in providing for the educational needs of the highly able were also examined. The study’s findings revealed crucial insights into teachers’ limited expertise in the field of serving highly capable students.

A thorough evaluation of Lebanon’s teachers’ instructional quality was conducted by the World Bank (2021), using 707 teachers from a nationally representative sample of the country’s schools. The report’s conclusions about the performance of the teachers demonstrated that Lebanon’s educational system still faces difficulties. According to Abdul-Hamid and Yassine (2020), memorizing was a typical teaching strategy used in public schools for all courses. This study may help to explain why Lebanese students performed poorly on application and reasoning questions that demanded the use of specific concepts to solve issues in international tests (Abdul-Hamid and Yassine, 2020; Antoun et al., 2023).

These findings can serve as a roadmap for effective legislative initiatives, teacher professional development programs, and other efforts to raise teaching standards among faculty members and, eventually, enhance student learning outcomes. A key component of raising the standard of education for all Lebanese students is developing policies that support effective teaching methods.

Since it is crucial for teachers to evaluate their practices in light of cultural differences and the role of gifted education in contributing to and/or aggravating socioeconomic injustices and inequities, professional development on culture and cultural diversity must be ongoing and substantive. Professional development opportunities should be provided for teachers to (1) understand the importance of their roles in identification, (2) address equity issues, and (3) learn concrete ways for supporting all their gifted students including the underserved ones in their classrooms.

## The conception of the proposed model

The proposed Lebanese model aimed to guide teachers who want to identify different types of gifted learners, including refugee learners, and make environments that are adequately challenging for them to reach their potential. Teachers must differentiate ability from talent to make it possible to identify a broad range of natural abilities and consider the many different ways that gifted students from various backgrounds may display their giftedness. Identifying a broad range of abilities helps with the identification of gifted underachievers, students from minority groups, and students with special needs. Through Gagné’s Differentiated Model of Giftedness and Talent (DMGT), teachers would be able to recognize the true potential of their students, regardless of their achievement level, which would help them support all kinds of gifted learners to reach their full potential. The approach might help in the creation of a precise scope and direction for offering appropriate and culturally significant programming, in a country with no specific reference for gifted education in its national curriculum (Sarouphim, 2009, 2015; El Khoury and Al-Hroub, 2018; Antoun et al., 2020). Overall, the development of a framework/model is also a valuable starting point that potentially supports Lebanon’s efforts to satisfy the requirements of its most academically gifted population, which in any context would appear to be a worthwhile goal. The model adopts Gagné’s (2008) talent development framework, getting past the idea that being gifted and having high cognitive capacity are interchangeable to understanding how talent in certain fields is discovered, nurtured, and developed through time with the aid of catalysts. Self-control, strategic risk-taking, and task persistence are considered crucial and complementary to strong academic and cognitive aptitude as well as creativity under this framework.

**Figure 1:**
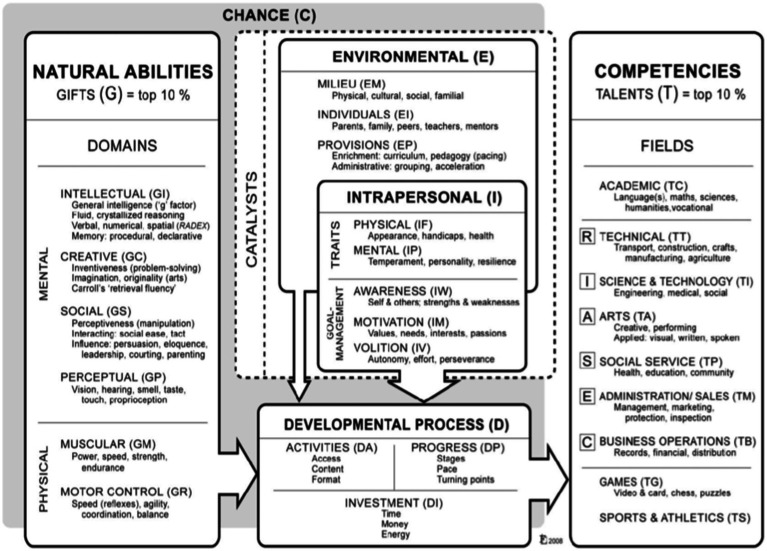
Gagné’s Differentiated Model of Giftedness and Talent (Gagné, 2008).

Examining the concept of giftedness and talent through the lens provided by Gagné (2008) reveals the nature and growth of human potentials and possibilities and that development is reliant upon the effect and influence of what Gagné defines in his model as catalysts, including intrapersonal and environmental factors and chance (See Figure 1). The model applies to any form of underrepresentation in talent development programs and extends to any country where the equity issue has been brought up (Gagné, 2011). According to Gagné (2008), the effect of culture is regarded as guiding every aspect of the developmental process. Therefore, it is necessary to consider the influence of culture as a differentiating factor in the way abilities are viewed within any specific culture. An understanding of the distinction between ability and talent might well assist teachers in identifying a broader range of natural abilities, and still fit within the Lebanese context, where different behaviors are valued and resourcing is limited. Furthermore, metacognitive awareness of the catalysts required for better progression through the developmental process may enhance the facilitation of the transformation of these abilities into competencies. This could assist Lebanese teachers to understand the importance of their contributing role as positive catalysts in the talent development process of their gifted and highly able students. Gagné highlighted the need of creating an engaging learning environment that gives children the chance to discover and nurture their abilities. He highlighted a growth perspective and stressed the significance of taking giftedness into account as a process-based entity embodied by numerous developmental chances.

Gagné’s (2008) DMGT indicates the diversity of human potentials and addresses the importance of the developmental process and intrapersonal and interpersonal factors in talent development helping students realize their giftedness. As Hymer (2012) argued, gifts are grown in an individual’s response to and interaction with his/her intellectual, physical, and social environment. All these relational parts of an integral whole contribute to talent development. Similarly, in Ziegler’s Actiotope Model of Giftedness, the environment should be in a position to support the process of the development of excellence. The focus of gifted education then shifts to helping all students reach their full potential by giving them a suitable context where opportunities for activating, exercising, and developing their gifts and becoming “talented” are abundant. This interactive nature between a learner and a learning environment is addressed (Lo et al., 2019). This occurs when an individual optimizes his/her abilities under the parameters set by society at a given time. Lo et al. (2019) use Mozart as an example of how his existent exceptional musical ability would not have been developed to accomplish such excellence without a context that provided conditions (e.g., a time/place that valued arts highly) conducive to developing Mozart’s musical ability.

The DMGT not only distinguished between potentials and accomplishments terminologically, but it also used that distinction as the basis for a theory of talent development, according to which, very simply put, high aptitudes, or the gifts, serve as the foundation for the progressive growth of abilities. First, it assumes that there are two different categories of human talents, namely, human potentialities that are separate from human accomplishments. In other words, the DMGT approach acknowledges and recognizes gifted underachievers. It also presupposes that these two categories of abilities may be evaluated and measured independently. Gagné’s DMGT model on giftedness aligns most closely with the Lebanese context where there are no formal identification procedures in place where some groups of gifted (gifted underachievers, gifted students in different domains other than the intellectual one) may fall through the cracks of the education system. Through DMGT, all types of gifted learners, including the gifted underachievers, are recognized and acknowledged. DMGT identifies all types of gifted learners and nurtures the potential in all students, which can play a key role in advocating for high-ability students who are less likely to be identified or in Lebanese schools– refugee students, students from financially disadvantaged families, and students of families who do not use English/French as the primary spoken language at home.

DMGT, compared to other western’s models such as Renzulli’s model, recognizes that gifted students have the potential to underachieve. When analyzing Renzulli’s model, it is apparent that his model does not address gifted underachievers. There are many reasons for gifted students to underperform at school and therefore, with this model, it would be difficult to support students if they are not even recognized as ‘gifted’. DMGT gives room to identify all types of intelligence, giving room for gifted refugees and marginalized populations to be identified.

Also, in DMGT’s model, different factors play a role in the actualization of students’ potential and their talent development. Teachers need to look at the factors through the lens of Gagné, trying to understand the underrepresentation of minorities and twice-exceptional population and their role in effectively identifying opportunities and experiences that promote their high ability student’s path toward achievement (Al-Hroub, 2014a).

Gifted education in Lebanon might not become soon a reality until the nation addresses larger economic, political, and social problems. However, in the meanwhile, we can improve by relying on a few of the best practices listed below.

### Identification using dynamic and alternative assessment

The field of gifted education has learned much about what constitutes a successful identification. However, there are a variety of variables that might influence how we identify talented individuals, some of which are beyond the control of educators. Some of the intelligence types could go undetected if potentially gifted students are diagnosed using standards like high intellectual achievement scores. Therefore, it is important to identify different types of intelligence using both subjective and objective measures.

In the United States, to determine a student’s placement in a gifted program, some states mandate the use of generic standardized examinations (Rinn et al., 2020). The sole use of standardized tests to determine giftedness excludes marginalized individuals and further distances them from their intellectual potential, which results in fewer students in these areas being labeled as gifted. Concerns about elitism have been raised since giftedness was adopted as a categorical notion based on psychometric cutoffs (Lupart and Webber, 2012; Porath, 2012; Al-Hroub, 2020). Identification can be difficult for students from diverse cultural, linguistic, and socioeconomic backgrounds. Educators need to consider the impact of out-of-school factors (e.g., harsh living conditions of low-income students) which can impede academic achievement. Those factors are referred to by Gagné (2008) as environmental catalysts. Gagné (2008) places less emphasis on identifying students who require special education based on their performance on intelligence tests and more emphasis on learning pathways that result in excellent performance for those who have the capability. Because it emphasizes the potential and expanding nature of human possibilities, this process-focused worldview easily resonates with a growth mindset perspective (Tirri, 2016). As a result, the concept of giftedness may be formatively studied. Identification can be difficult for students from diverse cultural, linguistic, and socioeconomic backgrounds, as their giftedness may show in non-psychometric ways, like inventiveness. Since Lebanon has no formal identification for the gifted population, the proposed multifaceted identification model, uses formal and informal, processes to properly identify and nurture gifted students with different abilities and backgrounds. In the end, group-specific norms have the best chance of boosting the equality of gifted education programs, especially when used in conjunction with local norms and universal screening (Peters and Engerrand, 2016). The multidimensional approach to identification combines psychometric and dynamic assessment (DA; Al-Hroub, 2012, 2021; Al-Hroub and Whitebread, 2019). DA is defined as an interactive diagnostic approach to conducting assessments that focuses on the ability of the learner to respond to intervention. Research studies highlight the importance of dynamic assessment in providing a more accurate assessment of learning potential and academic performance among various groups of disadvantaged students, such as students with low socioeconomic status, students from linguistic minorities or with migrant backgrounds, and students with disabilities. According to Al-Hroub (2009), this diagnostic approach takes into account the context of the learner and his/her learning experiences providing a means for assessing disadvantaged and underserved student populations. Since general IQ tests may undervalue the great potential of students who may have a learning disability or twice exceptional (e.g., gifted refugees) who are marginalized, a method is crucial for determining the untapped potential of students with varied profiles (Al-Hroub, 2012; El Khoury and Al-Hroub, 2018). Static psychometric tests are primarily interested in learned outputs, whereas DA approaches concentrate on learning processes and provide a more accurate and sensitive means of identifying diverse gifted children including those with learning disabilities (Al-Hroub, 2011). Psychometric tests have drawn more criticism for being inequitable to the underprivileged, the disadvantaged, the extraordinary, and the 2E (Al-Hroub, 2021). DA gives underprivileged students the chance to show off their learning potential. According to research, Dynamic assessment is considered one of the most promising practices in stimulating learning among various groups of students, including gifted and potentially gifted students (Popa and Pauc, 2015). It builds its foundation on Vygotskian concepts of sociocultural learning and zone of proximal development. This sheds light on the difference between a student’s performance and their possible performance after being assisted throughout a learning process by an experienced person. This indicates not only the role of catalysts (in the DMGT model) but also the importance of acknowledging the difference between a student’s potential level and his/her performance/achievement. DA emphasizes the interactional nature of learning, in line with Gagné’s DMGT model.

The research demonstrates that this approach has been particularly relevant for students from culturally and linguistically diverse backgrounds (Lidz and Elliott, 2006; Al-Hroub, 2011). According to Al-Hroub (2011, 2020), this approach can give a more complete picture of the student’s cognitive abilities and difficulties and also provide a means of assessing the potential development of underserved gifted students and of determining the discrepancy between potential and performance. He suggests obtaining important information from parents and teachers about their children’s academic and social activities, which may not be possible using standardized tests. DA employs several practices that integrate interaction with the child into the evaluation process and consider both the process and the outcome of learning. Application of DA in particular may allow us to differentiate between students who are at risk of not being identified as a result of poor instruction or inadequate literacy experience (Bridges and Catts, 2011). As minority students are seriously underrepresented and are less likely to do as well on traditional methods of gifted identification, they would benefit from flexible and non-traditional gifted identification procedures. This applies to underrepresented and marginalized gifted refugee children in Lebanon.

Sarouphim’s (2009) study of the use of DISCOVER, a performance-based evaluation, to identify gifted Lebanese students yielded positive outcomes. DISCOVER is one model for eliminating barriers and increasing facilitators in the identification of students from traditionally underrepresented gifted populations. The DISCOVER Assessment was created as a performance assessment using Gardner’s Multiple Intelligences and was promoted as being “bias-free” (Sarouphim, 1999, p. 246). Sarouphim’s (2009, 2015) two studies indicated that adopting DISCOVER as an assessment for identifying gifted Lebanese students might be promising for establishing programs for gifted students in Lebanon. The use of manipulatives and the option to administer the test in the children’s native tongue are only two of the features of DISCOVER that make it appropriate for use with populations that do not speak English as their first language, which makes it suitable for Lebanese and refugee children. Using DISCOVER, teachers collect students’ scores on all types of intelligence testing (verbal and nonverbal) which is also proposed by Al-Hroub (2013, 2014a). Another important factor to consider is identifying students who are under-challenged academically, socially, and creatively, reviewing the data to find those who have high potential and might not be performing at a higher level compared to aged peers as a result of demotivation. These identification procedures and instructional practices are the same for marginalized and non-marginalized gifted students.

Gagné’s (2008) DMGT model provides support for the identification process in terms of broadening the scope beyond high-achieving academic students. Teachers, parents, schools, and policymakers fit within Gagné’s Model as Environmental Catalysts, their contribution can be supportive or constraining in the process of talent development. If teachers commit to taking proactive measures to identify children—including those from marginalized groups—who need greater challenges, they can help all students reach their potential (Dixson et al., 2020).

DA comes in different forms, such as clinical interviews, formative assessments, project-based models, and test-intervene-test models (Al-Hroub, 2009). Below is an example of how the test-intervene-test dynamic assessment could be used with underrepresented gifted students in Lebanon.

***Step 1- Pretest:*** Teachers would identify students who demonstrate an aptitude for working in specific areas and are motivated to investigate a topic of interest. They would collect necessary data using a pre-test.

***Step 2- Teach:*** The teacher would design the intervention strategy and offer the necessary support during the intervention phase. They would observe target behaviors as signs of exceptional aptitude and differentiate instruction to capitalize on student ability.

***Step 3:* Posttest** -Teachers would test the students to identify students’ ability to benefit from instructional interventions such as differentiated activities.

## Instructional practices/provision

The provision of appropriate support requires a match between the perception of giftedness and related identification and programming. A teacher who perceives giftedness as a dynamic and multidimensional construct that develops depending on various factors would provide optimal learning environments that are appropriate and challenging to develop their gifted students’ potential. Inconsistencies in identification and subsequent programming may result in a program that fails to meet the needs, interests, or abilities of potential participants. For instance, if giftedness is viewed in terms of exceptionally high mathematical ability, then utilizing evaluation techniques that can pinpoint the possibility of greater performance in that specific area would be ideal. According to Renzulli (1986), there should be a logical connection between the definition and suggested identification and programming approaches. When designing instructional practices Lebanese teachers should differentiate between the characteristics of gifted students and those of talented ones. Research has warned of the harmful consequences of neglecting the needs of gifted students (Robinson et al., 2007; Rogers, 2007; Davis et al., 2011; Clark, 2014). It is also vital to learn about the characteristics of twice-exceptional learners (see Al-Hroub and Whitebread, 2008; Al-Hroub, 2009, 2021). Identified gifted students require curriculum and instructional strategies suitable for their higher level abilities, and if not provided, boredom and possibly underachievement may result.

Identifying learners’ academic demands is only useful if schools use it to meet those needs by giving each student the right amount of challenge. Every student must receive teaching that is properly challenging and culturally relevant for educators to deliver an equal education and for students to reach their full potential. Instead of expecting students to adjust to whatever services their schools happen to offer, schools must be willing to adapt to the variety of abilities, interests, and needs that students have (Dixson et al., 2020).

Acceleration, flexible grouping, differentiation, enrichment, and other services with empirical basis are offered to meet the academic needs of gifted learners. Since there is no legal requirement in Lebanon for gifted education, teachers are free to select any of these options, but they must carefully consider which options will benefit their students. Learning needs and barriers to learning may vary. Serving the needs of twice-exceptional is not the same as serving the needs of refugees; therefore, using a differentiated curriculum is recommended. A curriculum that is qualitatively differentiated takes into account students’ varying skills, interests, and learning profiles as well as the unique characteristics of gifted children (Maker and Shiever, 2010). Curriculum differentiation, according to researchers in the field of gifted education, entails designing and modifying the content, process, product, and learning environment (Tomlinson, 2003; Van Tassel-Baska, 2004; Maker and Shiever, 2010). It is critical to gauge a program’s effectiveness to decide if and how to make improvements. The differentiated program must be adjusted to meet the changing demands of learners in the Lebanese educational environment. The approach to take, according to Sarouphim (2015), is to “start small, analyze frequently, and update and expand according to demands” (p.209).

Findings from the literature support the notion that teachers in Lebanese primary schools appear to be very encouraging in the development of intrapersonal catalysts such as hard work, perseverance, and resilience (Antoun et al., 2020). However, if this form of encouragement could be extended into other areas, it may assist in fostering a broader range of talent development within Lebanese schools. As Hertzog (2017) stated, educators have a responsibility to be mindful and actively construct ideal circumstances for talent development because potential talent has to be nourished and developed in children.

Prejudice and stereotypes may contribute to segregated gifted and talented programs (Ford et al., 2020). Therefore, several issues need to be addressed when planning an effective gifted education provision: the current education system, pedagogy and assessment, complexity of definition, and teachers’ and parents’ attitudes and concerns. All interested parties, including educators, administrators, decision-makers, parents, and students themselves, must work together to implement gifted education to enhance learning. Efforts to maximize learning will be fragmented and ineffective unless adjustments are made in each of these areas.

### Professional learning

A re-examination of conventional theory and practice in spotting gifted students and attending to the needs of individuals whose abilities frequently go undetected, uncelebrated, and unserved is needed. The latest Lebanese literature has several significant implications for the provision of gifted education in Lebanon’s future and could serve as a useful starting point for teacher education programs and professional development for working teachers. In light of the research findings, it is clear that talented children in Lebanon do not benefit from the full spectrum of evidence-based educational modifications that have been proven most effective in western nations. Antoun et al. (2020) noted that instructors in Lebanon had no formal training in gifted education and were instead influenced by a traditional understanding of school culture. However, establishing critical pedagogy can change the current school system in Lebanon, and indeed in other countries with similar conceptualizations that privilege achievement with little attention given to the intrinsic value of natural ability.

To be able to recognize the various traits of exceptional learners, differentiate between potential and achievement, and be responsive to gifted students’ diverse needs, teachers need to have proper professional development to help transform gifted potential into outstanding performance (Al-Hroub, 2007).

El Khoury and Al-Hroub (2018) found that a large number of participant teachers in their study stated that they had never given giftedness any thought. An important finding from different studies is that gifted students in Lebanon do not appear to be receiving educational accommodations underpinned by a sound research evidence base (Sarouphim, 2010; Antoun et al., 2020; Al-Hroub, 2022). This paper highlights the need for education for teachers in optimal ways of working with gifted students. To support the education and development of their exceptional children, all teachers need to have some level of professional preparation. Professional learning should be holistic taking into account the learner’s academic, social, and emotional needs. It should also prepare educators to be empowered to use varied methods of identification, curriculum modification, and instruction.

To give instructors continuous learning opportunities, a professional development plan for gifted education should be established. This has implications for how courses are designed and for university professors in terms of what to cover in their teacher preparation courses. At the same time, teachers need to be provided with adequate education, time, and support to be able to appropriately provide for highly able learners at their respective schools. As a basis for these measures, a sound Lebanese policy on gifted education would need to be developed by the policymakers and the Ministry of Education.

### Community engagement

To facilitate the aforementioned changes and to prepare for the implementation of a gifted program, awareness campaigns and community engagement are considered necessary. Community engagement techniques can involve sharing information or eliciting opinions, and effectively involving people in planning, decision-making, and evaluation. This, along with specific professional development would help create the opportunity for teachers and parents to make valuable contributions to the evaluation process of the effectiveness and efficiency of the gifted program.

Ultimately, the current paper has provided valuable insights into the essential steps required to effectively provide for gifted students in Lebanon. Realizing the issues about gifted education appropriately is necessary before establishing any related policies. The current findings may serve as a wake-up call and indicate that it is time for more of us to act with renewed vigor to implement tangible reforms to gifted identification processes and programming. The model could be used by the Lebanese Ministry of Education to assist in creating a strategy for the education of all gifted children, including the marginalized ones. At the national level, policymakers should enact laws requiring the inclusion of gifted education in teacher preparation programs.

In conclusion, it is important to Lebanon as well as to the individual teachers to ensure that the country’s young people are educated in ways that will allow them to reach their potential and be in the best position to positively contribute to Lebanese society. This framework is intended to serve as a roadmap for policymakers, educators, and professional learning experts who make decisions in teacher preparation programs. Gifted education is a need, not a privilege to a few racial, economic or cultural groups. Lebanon is in needs to strive to serve students equitably and make access to gifted education and talent development programs for all our students. Now is the time for a change.

### Limitations of the proposed model

The model proposes guided support when teaching any new skill which requires proficient teachers who can help in the process. This might be problematic if teachers in Lebanon are not provided with professional knowledge and needed resources. Also, the learner might be very dependent on guided information which makes him/her less likely to explore ways of problem-solving.

### Implications and limitations

As research in gifted education is still in its inception phase in Lebanon, continued research evaluating the proposed model and investigating factors that contribute to the success of gifted students, including those from underprivileged backgrounds would add to the scope of the Lebanese literature. More research on refugee gifted populations is needed to paint a true picture of ‘giftedness’, as lack of data remains a constraint to differentiated strategies. There is also a need to explore and understand the support structures and educational opportunities present for gifted students in different Lebanese schools across the country, as it serves as the basis of intervention strategies and informs teacher training, as they relate to gifted education.

## Author contributions

The author confirms being the sole contributor of this work and has approved it for publication.

## Conflict of interest

The author declares that the research was conducted in the absence of any commercial or financial relationships that could be construed as a potential conflict of interest.

## Publisher’s note

All claims expressed in this article are solely those of the authors and do not necessarily represent those of their affiliated organizations, or those of the publisher, the editors and the reviewers. Any product that may be evaluated in this article, or claim that may be made by its manufacturer, is not guaranteed or endorsed by the publisher.
